# Respiratory Commensal Bacteria Increase Protection against Hypermucoviscous Carbapenem-Resistant *Klebsiella pneumoniae* ST25 Infection

**DOI:** 10.3390/pathogens11091063

**Published:** 2022-09-19

**Authors:** Stefania Dentice Maidana, Ramiro Ortiz Moyano, Juan Martin Vargas, Kohtaro Fukuyama, Shoichiro Kurata, Vyacheslav Melnikov, María Ángela Jure, Haruki Kitazawa, Julio Villena

**Affiliations:** 1 Laboratory of Immunobiotechnology, Reference Centre for Lactobacilli (CERELA-CONICET), San Miguel de Tucumán 4000, Argentina; 2 Laboratory of Antimicrobials, Institute of Microbiology “Luis C. Verna”, Faculty of Biochemistry, Chemistry and Pharmacy, National University of Tucuman, San Miguel de Tucumán 4000, Argentina; 3 Food and Feed Immunology Group, Laboratory of Animal Food Function, Graduate School of Agricultural Science, Tohoku University, Sendai 980-8572, Japan; 4 Laboratory of Molecular Genetics, Graduate School of Pharmaceutical Sciences, Tohoku University, Sendai 980-8578, Japan; 5 Gabrichevsky Research Institute for Epidemiology and Microbiology, 125212 Moscow, Russia; 6 Livestock Immunology Unit, International Education and Research Centre for Food and Agricultural Immunology (CFAI), Graduate School of Agricultural Science, Tohoku University, Sendai 980-8572, Japan

**Keywords:** respiratory commensal bacteria, respiratory immunity, next generation probiotics, *Corynebacterium pseudodiphtheriticum*, *Klebsiella pneumoniae*, antibiotic resistance, immunobiotic

## Abstract

In a previous work, we demonstrated that nasally administered *Corynebacterium pseudodiphtheriticum* 090104 beneficially modulated the respiratory innate immune response and improved the protection against Respiratory Syncytial Virus and *Streptococcus pneumoniae* in mice. In this work, we aimed to evaluate whether the immunomodulatory 090104 strain was able to enhance the resistance against the respiratory infection induced by hypermucoviscous carbapenemase-producing (KPC-2) *Klebsiella pneumoniae* strains belonging to the sequence type (ST) 25. The nasal treatment of mice with *C. pseudodiphtheriticum* 090104 before the challenge with multiresistant *K. pneumoniae* ST25 strains significantly reduced lung bacterial cell counts and lung tissue damage. The protective effect of the 090104 strain was related to its ability to regulate the respiratory innate immune response triggered by *K. pneumoniae* challenge. *C. pseudifteriticum* 090104 differentially modulated the recruitment of leukocytes into the lung and the production of TNF-α, IFN-γ and IL-10 levels in the respiratory tract and serum. Our results make an advance in the positioning of *C. pseudodiphtheriticum* 090104 as a next-generation probiotic for the respiratory tract and encourage further research of this bacterium as a promising alternative to develop non-antibiotic therapeutical approaches to enhance the prevention of infections produced by microorganisms with multiple resistance to antimicrobials such as KPC-2-producing hypermucoviscous *K. pneumoniae* strains belonging to ST25.

## 1. Introduction

Studies carried out in Argentina from the year 2006 demonstrated a remarkable dissemination of carbapenemase-producing (KPC-2) *Klebsiella pneumoniae* belonging to the sequence type (ST) 258 [1,2]. However, more recent studies allowed the detection of an increase in KPC-2-producing hypermucoviscous *K. pneumoniae* strains belonging to ST25, which have been associated with nosocomial infections [3]. In this sense, our previous studies have shown that KPC-2-producing *K. pneumoniae* is endemic in hospitals in the province of Tucuman and, in accordance with observations in other regions of Argentina, in the northwest we have recently detected the spread of hypermucoviscous KPC-2-producing *K. pneumoniae* ST25 [4,5]. Our findings demonstrated that the urinary and respiratory tracts were the most common sources of clinical samples with carbapenem-resistant *K. pneumoniae* strains, followed by soft tissues and blood [4,5]. Furthermore, we have reported that some of these *K. pneumoniae* strains possess virulence factors found in other strains that have been shown to be hypervirulent [6]. The high incidence of the ST25 clone in our region associated with respiratory and systemic infections emphasizes the importance of implementing epidemiological, genetic, and phenotypic studies of this particular ST. Moreover, considering the high capacity of hypermucoviscous KPC-2-producing *K. pneumoniae* ST25 to resist antimicrobial treatments, it is necessary to search for new alternatives to reduce the incidence of infections caused by these pathogens.

Probiotics have been proposed to increase the resistance to respiratory infections. In the last decade, great advances have been made in the characterization of the beneficial effect of probiotic microorganisms in the prevention of infections caused by respiratory pathogens such as *Streptococcus pneumoniae* [7,8], influenza virus (IFV) [9] and respiratory syncytial virus (RSV) [10,11]. However, the effect of probiotics against multiresistant pathogens that infect the respiratory tract such as *K. pneumoniae* has not been investigated in detail. Using in vitro approaches or mice models of multiresistant *K. pneumoniae* intestinal colonization, it was shown that *Lactobacillus* spp. strains are able to reduce the pathogen colonization and biofilm formation [12,13,14,15]. The ability of lactobacilli to inhibit the colonization of *K. pneumoniae* was associated with organic acid production [14,15] and the secretion of proteins with potential antimicrobial activity [16,17]. The possibility of modulating the immune system to improve resistance to *K. pneumoniae* infection has not been studied in depth. To the best of our knowledge, only one study has reported the ability of immunomodulatory probiotics to enhance resistance to *K. pneumoniae* infection [18]. It was shown that the oral administration of *L. plantarum* CIRM653 to mice significantly reduced the lung inflammatory damage induced by *K. pneumoniae* nasal challenge.

The potential beneficial effect of respiratory commensal bacteria against lung infections was recently reported by our group using mice models of pneumococcal and RSV infections [19,20,21]. In our hands, the nasal administration of *Corynebacterium pseudodiphtheriticum* 090104 or *Dolosigranulum pigrum* 040417 to mice differentially modulated the respiratory innate immune response, allowing an improved response to RSV or *S. pneumoniae*. However, the effect of immunomodulatory respiratory commensal bacteria on the resistance to a Gram-negative bacterial pathogen was not investigated before. Then, in this work we aimed to evaluate whether the immunomodulatory strain *C. pseudodiphtheriticum* 090104 was able to modulate the innate respiratory immune response and enhance the resistance to hypermucoviscous KPC-2-producing *K. pneumoniae* ST25.

## 2. Materials and Methods

### 2.1. Microorganisms

*Corynebacterium pseudodiphtheriticum* 090104 was cultured at 37 °C for 18 h (late log phase) in trypticase soy broth. Bacteria suspensions were prepared as previously described [19]. Briefly, the bacteria were harvested by centrifugation at 3000× *g* for 10 min, washed three times with sterile 0.01 M phosphate buffer saline (PBS, pH 7.2) and suspended in sterile PBS.

*K. pneumoniae* LABACER 01 and LABACER 27 were isolated at the “Angel Cruz Padilla” hospital in the city of San Miguel de Tucuman (Tucuman, Argentina). Both hypermucoviscous carbapenem-resistant *K. pneumoniae* ST25 strains were isolated from the intensive care unit and were selected based on their virulent capacity [4,5]. The LABACER 01 strain was recovered from a bone sample of a 20-year-old male patient and the LABACER 27 strain was isolated from the lung sample of a 63-year-old male patient. Both microorganisms were identified by matrix-assisted laser desorption/ionization (MALDI-TOF) (Microflex LT; Bruker Daltonik GmbH, Bremen, Germany) and kept in the Culture Collection of the Certified Bacteriology Laboratory (LABACER, National University of Tucuman, Tucuman, Argentina) [4,6]. The hypermucoviscous phenotype of *K. pneumoniae* LABACER 01 and LABACER 27 was determined by the string test [20].

### 2.2. Murine Infection Model

All experiments were performed in accordance with the guide for the care and use of laboratory animals and approved by the CERELA-CONICET Animal Care and Ethics Committee under the BIOT-CRL/19 protocol. Six-week-old male BALB/c mice were used, obtained from the closed colony maintained in the CERELA Animal Facility (Reference Center for Lactobacilli, CONICET, Tucuman). The animals were housed in plastic cages at room temperature and fed a conventional balanced diet ad libitum. A total of 5–6 mice per group were used for each time point tested.

### 2.3. Respiratory Infection with K. pneumoniae LABACER 01 and LABACER 27

*C. pseudodiphtheriticum* 090104 was nasally administered to mice for 5 consecutive days at a dose of 10^8^ cells/mouse/day in 50 µL of PBS. Control animals only received 50 µL of PBS. The treated group and the untreated control mice were fed a conventional balanced diet ad libitum. One day after the last administration of the 090104 strain, these mice and controls were lightly anesthetized and dropwise administered, through the nostrils, 100 uL of sterile PBS containing 10^7^ CFU of *K. pneumoniae* LABACER 01 or LABACER 27. The infective dose selected for this work arose from experiments in which doses between 10^5^ and 10^9^ CFU of *K. pneumoniae* LABACER 01 or LABACER 27 were evaluated [21].

### 2.4. K. pneumoniae Counts in Lungs

*K. pneumoniae* LABACER 01 or LABACER 27 cell counts in the lungs were performed in mice sacrificed on day 2 post-infection and their lungs were excised, weighed and homogenized in 5 mL of sterile peptone water. Homogenates were appropriately diluted in BHI broth, plated in duplicate on blood agar and incubated for 18 h at 37 °C. *K. pneumoniae* colonies were counted and results were expressed as log_10_ CFU per gram of lung. Hemocultures were performed similarly, and results were expressed as positive or negative.

### 2.5. Lung Damage

Albumin content, as a measure to quantify the increased permeability of the alveolar-capillary barrier, and the activity of the intracellular enzyme lactate dehydrogenase, an indicator of cellular cytotoxicity, were determined in BAL samples [22,23]. Albumin content was determined colorimetrically based on albumin binding to bromocresol green using a Wiener-Lab albumin diagnostic kit. LDH activity, expressed as units per liter of BAL, was determined by measuring the formation of the reduced form of NAD^+^ using Wiener’s reagents and procedures (Wiener-Lab).

### 2.6. Leukocytes Counts

The total number of BAL and blood leukocytes was determined using a haemocytometer. Differential cell counts in BAL and blood were assessed by microscopically counting cells in smears stained with May-Grunwald-Giemsa as described previously [19,22].

### 2.7. Serum Cytokines and Bronchoalveolar Lavages

Blood samples were obtained by cardiac puncture and collected in heparinized tubes [22,23]. BAL samples were obtained according to the technique developed in the Laboratory Immunobiotechnology of CERELA-CONICET (San Miguel de Tucuman, Argentina) [19]. The trachea was exposed and intubated with a catheter, then two sequential bronchoalveolar lavages were performed on each mouse by injecting sterile PBS. The recovered fluid was centrifuged for 10 min at 900× *g* and the fluid was frozen at −70 °C for subsequent cytokine determinations. Tumor necrosis factor alpha (TNF-α), interferon gamma (IFN-γ) and interleukin 10 (IL-10) concentrations were determined in serum and BAL using commercial ELISA kits. IFN-γ (Mouse IFN-gamma Quantikine ELISA Kit, sensitivity: 2 pg/mL) and IL-10 (Mouse IL-10 Quantikine ELISA Kit, sensitivity: 5.2 pg/mL) were from R&D Systems (Minneapolis, MN, USA). TNF-α (Mouse TNF alpha ELISA Kit, sensitivity: 9.1 pg/mL) was from Abcam (Cambridge, UK).

### 2.8. Statistical Analysis

The experiments were performed in triplicate and the results were expressed as mean ± SD. Statistical analyses were performed using Prism 8.0 (GraphPad software, San Diego, CA, USA). Comparisons among multiple groups across multiple time points were performed using a two-way ANOVA with Tukey’s multiple comparison post hoc test. Comparisons between two groups were performed using unpaired Student’s *t*-tests. Differences were considered significant at *p* < 0.05.

## 3. Results and Discussion

**Figure 1:**
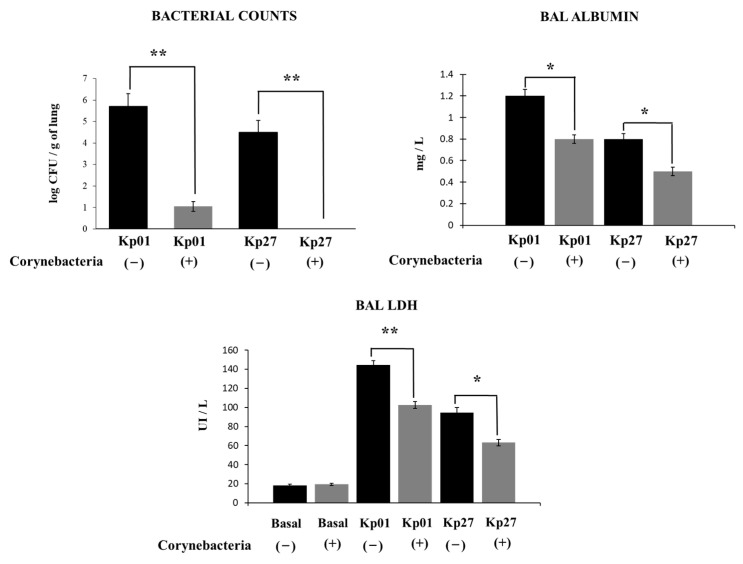
Effect of *Corynebacterium pseudodiphtheriticum* on lung colonization and damage induced by KPC-2-producing hypermucoviscous ST25 strains of *Klebsiella pneumoniae*. Immunocompetent adult BALB/c mice (6 weeks) were nasally stimulated with *C. pseudodiphtheriticum* 090104 for three days and then challenged nasally with *K. pneumoniae* LABACER 01 (Kp01) or LABACER 27 (Kp27). Two days after challenge, bacteria cell counts in lung homogenates, lactate dehydrogenase (LDH) enzyme activity and albumin concentration were determined in broncho-alveolar lavages (BAL). Results represent data from three independent experiments. Asterisks indicate significant differences between the indicated groups, (*) *p* < 0.05, (**) *p* < 0.01. Basal levels of BAL albumin were below the detection limit.

For the experiments, two KPC-2-producing hypermucoviscous *K. pneumoniae* ST25 strains were selected based on their virulent capacity [6]. The intranasal challenge of mice with both *K. pneumoniae* LABACER 01 or LABACER 27 led to significant bacterial burden in the lungs after two days of infection (Figure 1). The lung bacterial counts in mice infected with the LABACER 01 strain were significantly higher than animals infected with the LABACER 27 strain (Figure 1). In addition, blood bacterial cultures showed that only *K. pneumoniae* LABACER 01 was able to spread from the lungs, since blood cultures in the LABACER 27 group were negative (data not shown). Interestingly, the nasal treatment of mice with *C. pseudodiphtheriticum* 090104 before the challenge with the KPC-2-producing hypermucoviscous *K. pneumoniae* ST25 strains significantly reduced lung bacterial cell counts (Figure 1). The treatment with the respiratory commensal bacterium was able to inhibit the lung colonization of *K. pneumoniae* LABACER 27 (Figure 1) and to avoid the dissemination into blood of *K. pneumoniae* LABACER 01 (data not shown).

We also evaluated the lung damage induced by the infections with the KPC-2-producing hypermucoviscous *K. pneumoniae* ST25 strains by the study of biochemical parameters in BAL samples. As shown in Figure 1, both *K. pneumoniae* LABACER 01 and LABACER 27 significantly increased albumin concentration and LDH activity in BAL samples compared to non-infected controls, indicating that these pathogens produce cell damage in the lungs and increase the permeability of the alveolar-capillary barrier. As expected, it was also observed that the levels of LDH and albumin in BAL samples were significantly higher in the LABACER 01 group than in mice infected with *K. pneumoniae* LABACER 27 (Figure 1). The nasal priming of mice with *C. pseudodiphtheriticum* 090104 significantly reduced the levels of the biochemical parameters evaluated in BAL samples, indicating that the respiratory commensal bacterium is able to reduce the lung tissue damage induced by the KPC-2-producing hypermucoviscous *K. pneumoniae* ST25 strains infections (Figure 1). These results together indicate that *C. pseudodiphtheriticum* 090104 is an interesting alternative to increase the resistance to colonization and reduce lung damage caused by the respiratory infection with *K. pneumoniae* ST25 strains.

Of note, we used 10^7^ CFU of KPC-2-producing hypermucoviscous *K. pneumoniae* ST25 strains to induce respiratory infections in adult immunocompetent mice. Our findings are in line with other studies carried out in mice, in which doses of 10^7^–10^8^ CFU of *K. pneumoniae* clinical isolates were needed to infect immunocompetent animals. In this regard, experiments carried out in immunocompetent mice showed that doses of 10^7^ CFU or higher are necessary to achieve a respiratory infection that reaches densities of 6–7 log_10_ CFU/g lung when the clinical isolates *K. pneumoniae* KPC^+^ or OXA-48^+^ are used [24]. Similarly, doses of 10^8^ CFU were required to achieve respiratory infections in adult C57BL/6 mice with clinical isolates of *K. pneumoniae* [25]. It should be noted that other *K. pneumoniae* strains are highly virulent and, therefore, fatal respiratory infections can be achieved with doses lower than those used with clinical isolates. One of the most widely used strains for such studies is *K. pneumoniae* ATCC 43816 as it recapitulates acute pneumonia with fatal systemic spread at a relatively low infectious dose (10^4–^10^5^ CFU intranasally) when administered to adult C57BL/6 mice [26,27,28]. It has been shown that *K. pneumoniae* ATCC 43,816 is also capable of producing lethal pneumonia in BALB/c mice when administered intranasally at a dose of 10^4^ CFU [29,30]. The different ability of *K. pneumoniae* strains to efficiently infect the respiratory tract of mice could be related to the set of virulence factors coded in their genomes. In fact, our recent comparative genomic analysis showed that in the LABACER strains, *ybt* or *iuc* genes were not detected [6], which code for yersinibactin and aerobactin, respectively [31]. The siderophore ybt is sufficient to promote pneumonia in mice experimental models and it has been associated with respiratory tract infections in patients [32,33]. In addition, experiments with aerobactin-producing and non-producing *K. pneumoniae* strains showed that mice challenged with *K. pneumoniae* iuc^+^ had lower survival and greater lung tissue damage compared to animals infected with the iuc^−^ strain [34]. Then, the absence of these virulence genes in the LABACER strains would explain why higher bacterial doses (10^7^ CFU) are necessary to infect the respiratory tract of mice compared to other *K. pneumoniae* such as ATCC 43,816 [34]. These results raise an important question regarding the ability of *C. pseudodiphtheriticum* 090104 to protect against infections caused by *K. pneumoniae*, since its effect could only be observed with less virulent strains. Further studies evaluating the capacity of *C. pseudodiphtheriticum* 090104 to protect against highly virulent *K. pneumoniae* strains such as ATCC 43,816 would be of great importance to fully characterize their ability to protect the respiratory tract.

**Figure 2:**
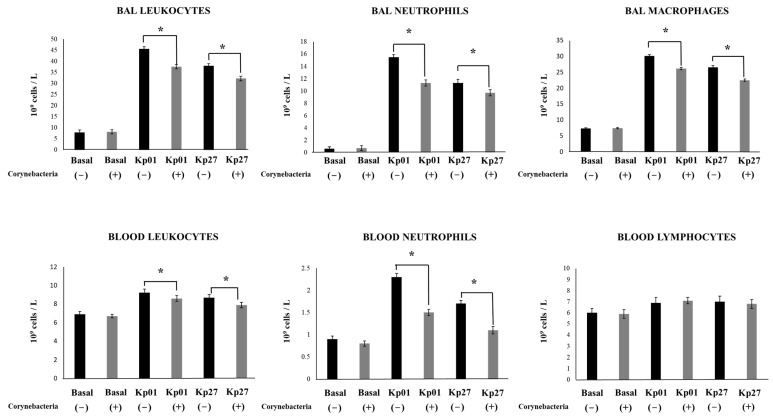
Effect of *Corynebacterium pseudodiphtheriticum* on respiratory and blood leukocytes counts induced by KPC-2-producing hypermucoviscous ST25 strains of *Klebsiella pneumoniae*. Immunocompetent adult BALB/c mice (6 weeks) were nasally stimulated with *C. pseudodiphtheriticum* 090104 for three days and then challenged nasally with *K. pneumoniae* LABACER 01 (Kp01) or LABACER 27 (Kp27). Two days after challenge, total and differential leukocytes counts were determined in broncho-alveolar lavages (BAL) and blood. Results represent data from three independent experiments. Asterisks indicate significant differences between the indicated groups, (*) *p* < 0.05.

**Figure 3:**
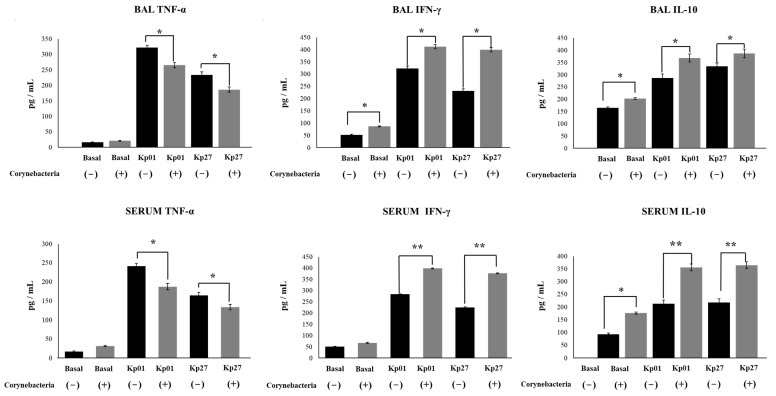
Effect of *Corynebacterium pseudodiphtheriticum* on respiratory and serum cytokines induced by KPC-2-producing hypermucoviscous ST25 strains of *Klebsiella pneumoniae*. Immunocompetent adult BALB/c mice (6 weeks) were nasally stimulated with *C. pseudodiphtheriticum* 090104 for three days and then challenged nasally with *K. pneumoniae* LABACER 01 (Kp01) or LABACER 27 (Kp27). Two days after challenge, TNF-α, IFN-γ and IL-10 levels were determined in broncho-alveolar lavages (BAL) and serum. Results represent data from three independent experiments. Asterisks indicate significant differences between the indicated groups, (*) *p* < 0.05, (**) *p* < 0.01.

We also aimed to investigate whether the effect of *C. pseudodiphtheriticum* 090104 in the resistance against *K. pneumoniae* infection was related to the ability of the respiratory commensal bacterium to modulate innate immunity. Thus, we evaluated the changes in BAL and blood leukocytes (Figure 2) and BAL and serum cytokines (Figure 3) on day 2 post-infection.

The differential counts of BAL leukocytes showed that the infection with both *K. pneumoniae* LABACER 01 and LABACER 27 increased the values of leukocytes, neutrophils and macrophages in the respiratory tract when compared to non-infected mice (Figure 2). Similarly, the infection enhanced the levels of blood leukocytes, neutrophils, and lymphocytes. Significant differences were found between the *K. pneumoniae* LABACER 01- and LABACER 27-infected groups when comparing BAL and blood leukocyte counts. These differences were mainly due to the changes in neutrophils counts since mice challenged with *K. pneumoniae* LABACER 01 presented levels of these cells that were significantly higher than those of mice infected with the LABACER 27 strain (Figure 2). We also observed that the intranasal challenge with the KPC-2-producing hypermucoviscous *K. pneumoniae* ST25 strains induced a marked increase in the levels of the inflammatory cytokines TNF-α and IFN-γ in both BAL and serum samples compared to uninfected controls (Figure 3). As expected, the LABACER 01 strain was able to trigger a higher increase in BAL and serum TNF-α and IFN-γ than *K. pneumoniae* LABACER 27. We also evaluated the levels of the regulatory cytokine IL-10, which was increased in both serum and BAL samples after the challenge with the respiratory pathogens (Figure 3).

Studies have demonstrated that the respiratory challenge of mice with *K. pneumoniae* strains trigger potent inflammatory responses. It was shown that adult C57BL/6 mice increase the levels of TNF-α and IL-6 in the respiratory tract after the nasal challenge with *K. pneumoniae* ATCC 43,816 [35]. Subsequent studies demonstrated that CCR2^+^ monocytes produce TNF-α in the infected lungs, which trigger the pulmonary infiltration neutrophils in response to nasal challenge with the ATCC 43,816 strain [26]. Increases in the pulmonary levels of IL-1β, TNF-α, IL-6 [25], MCP-1, MIP-2 and CXCL1 [36] as well as neutrophils [25,36] have been observed in mice infected with clinical isolates of *K. pneumoniae*. Our studies are in line with these previous reports since the KPC-2-producing hypermucoviscous *K. pneumoniae* ST25 strains increased the levels of neutrophils, macrophages, TNF-α and IFN-γ in the respiratory tract and blood, with the effect of LABACER 01 being more marked than that of LABACER 27.

Our results also showed that the treatment of mice with *C. pseudodiphtheriticum* 090104 did not induce changes in the number of BAL or blood leukocytes before challenge with pathogens (Figure 2). Interestingly, animals treated with the 090104 strain had significantly lower counts of BAL leukocytes, neutrophils, and macrophages than their respective infected controls after the infection with *K. pneumoniae*. In addition, the counts of blood leukocytes and neutrophils were lower in mice treated with *C. pseudodiphtheriticum* 090104 than in control animals infected with *K. pneumoniae* LABACER 01 or 27. No differences were observed in the number of blood lymphocytes when 090104-treated and non-treated groups were compared (Figure 2). The nasal priming of mice with *C. pseudodiphtheriticum* 090104 did not induce changes in the levels of BAL or serum cytokines before challenge with LABACER 01 or 27 with the exception of increases in BAL IFN-γ, and serum and BAL IL-10 (Figure 3). Of note, mice treated with the 090104 strain had significantly lower levels of BAL and serum TNF-α and higher concentrations of IFN-γ and IL-10 than controls after the challenges with *K. pneumoniae* LABACER 01 or 27 (Figure 3). Thus, these results together indicate that *C. pseudodiphtheriticum* 090104 is able to differentially modulate the respiratory and systemic innate immune response against *K. pneumoniae* ST25 strains. Of note, the nasal priming with *C. pseudodiphtheriticum* 090104 was able to diminish the increases of blood and BAL neutrophils, macrophages, TNF-α and IFN-γ. Furthermore, the respiratory commensal bacterium increased the levels of IL-10, indicating its ability to differentially regulate the inflammatory response.

Studies in mice demonstrated that the inflammatory response could have both protective and detrimental roles in resistance to respiratory infection by *K. pneumoniae* [27,29,31,35]. It was reported that C57BL/6 adult mice treated with antibiotics have alveolar macrophages with significantly decreased bactericidal activities and reduced capacity to produce IL-6 and TNF-α compared to controls [35]. Of note, antibiotic-treated animals are more susceptible to the infection with *K. pneumoniae* ATCC 43,816 compared to control mice without antibiotic therapy. It was also demonstrated that the treatment of experimental animals with anti-Ly6G monoclonal antibodies, which induces neutropenia in mice, significantly increases susceptibility to pulmonary infection by *K. pneumoniae* [31]. These results clearly indicate that the inflammatory response is necessary to control the colonization of *K. pneumoniae* in the respiratory tract. In contrast, it was shown that a combined antibiotic treatment can protect mice from severe pneumonia induced by *K. pneumoniae* ATCC 43,816 and that this beneficial effect is associated with the reduction of the accumulation of inflammatory cells in the lungs [27]. Antibiotic treatment decreased the abnormally elevated levels of TNF-α, IL-1β and IL-6, increasing survival of infected mice. It was also reported that CD36^−/−^ adult C57Bl/6J mice, which possess macrophages with lower phagocytic activity as well as a decreased ability to produce IFN-γ, IL-1β, IL-6 and TNF-α, are highly susceptible to the respiratory infection with hypermucoviscous *K. pneumoniae* [37]. Of note, CD36^−/−^ mice infected with *K. pneumoniae* showed significantly higher levels of lung neutrophil infiltration as well as lower levels of the regulatory cytokine IL-10. Together, these findings indicate that protection against *K. pneumoniae* respiratory infection could be achieved by inducing an inflammatory response capable of eliminating the pathogen, which must be efficiently regulated to prevent inflammatory damage. Thus, the results of this work allow us to speculate that the nasal priming with *C. pseudodiphtheriticum* 090104 can help to beneficially regulate the inflammatory response allowing a lower lung tissue damage together with an improved clearance of the pathogens from both the respiratory tract and blood.

We demonstrated previously that *C. pseudodiphtheriticum* 090104 stimulated the production of IFN-β and IFN-γ in CD45 ^+^ SiglecF^+^ alveolar macrophages after the activation of TLR3 in the respiratory tract by the nasal administration of poly(I:C) or RSV challenge [19]. In addition, in a mice model in which a mixture of the TLR2 ligands MALP2 and Pam3CSK4 was used to mimic the respiratory inflammatory response induced by Gram-positive pathogens, we demonstrated that the 090104 strain enhanced the levels of IFN-γ, IFN-β and IL-10 and protected animals from the TLR2-mediated lung injury [22]. Furthermore, we showed that the differential immune response induced by *C. pseudodiphtheriticum* 090104 was related to its ability to modulate the activation of alveolar macrophages [23]. Therefore, it is tempting to speculate that the nasal priming with the respiratory commensal bacterium modulates the activity of alveolar macrophages, inducing a differential immune response coordinated by a combination of cytokines in such a way that respiratory pathogen clearance is enhanced but inflammation is simultaneously better controlled. In this way, the respiratory commensal bacterium would modulate mechanisms of innate immunity that are common as the first line of defense against a wide variety of microorganisms and in such way that would increase the protection against viral Gram-positive and Gram-negative pathogens.

## 4. Conclusions

It was reported that respiratory commensal bacteria are able to prevent the colonization of pathogens by obstructing adhesion sites, competing for nutrients, producing antimicrobial substances and modulating the immune system of the host [22,23,38]. In this regard, we reported previously that *C. pseudodiphtheriticum* 090104 modulates respiratory immunity, increasing the protection against viral [19] and Gram-positive [22] pathogens. In this work, we have advanced in the characterization of the beneficial properties of the 090104 strain by studying its capacity to modulate the respiratory innate immune response triggered by a Gram-negative pathogen. Our results make an advance in the positioning of *C. pseudodiphtheriticum* 090104 as a next-generation probiotic for the respiratory tract and encourage further research of this bacterium as a promising alternative to develop non-antibiotic therapeutical approaches to enhance the prevention of infections produced by microorganisms with multiple resistance to antimicrobials such as KPC-2-producing hypermucoviscous *K. pneumoniae* strains belonging to ST25.

## Data Availability

Data are contained within the article.
